# Supplementary material for: ProteinShader: illustrative rendering of macromolecules
Source: BMC Struct Biol. 2009 Mar 30;9:19. doi: 10.1186/1472-6807-9-19 (PMC2672931; doi:10.1186/1472-6807-9-19)
Supplement: Additional file 1 — ProteinShader program without source code. This compressed file contains the complete ProteinShader program including associated libraries, but no source code. A README.txt file gives an overview of the ProteinShader distribution, and the index.html file in the help subdirectory has directions on getting started with the program as well as a set of tutorials. [file 1472-6807-9-19-S1.zip › ProteinShader-beta-0_9_4-binary/help/api/org/proteinshader/structure/Heterogen.html]

Heterogen (ProteinShader API)


|  |  |  |
| --- | --- | --- |
| **Overview** \| **Package** \| **Class** \| **Use** \| **Tree** \| **Deprecated** \| **Index** \| **Help** | |  |
| **PREV CLASS**   **NEXT CLASS** | **FRAMES**    **NO FRAMES**     **All Classes** |  |
| SUMMARY: NESTED \| FIELD \| CONSTR \| METHOD | DETAIL: FIELD \| CONSTR \| METHOD |  |


---


## org.proteinshader.structure Class Heterogen

```
java.lang.Object
  org.proteinshader.structure.Residue
      org.proteinshader.structure.Heterogen
```

**All Implemented Interfaces:**: IDTest, Visitable

---

``` public class Heterogen extends Residue ```

This concrete subclass of the abstract class Residue stores
information on a heterogen in a PDB structure entry. Because water
molecules are a special case of the heterogen concept, their
information is stored in a Water object rather than in a Heterogen
object.

---

| **Field Summary** | |
| --- | --- |
| `static String` | `HETEROGEN_PREFIX`             'HET\_' will be added to the beginning the residueID for a Heterogen (if it is not already present). |


**Constructor Summary**

`Heterogen(String name,
String residueID,
String chainID,
String modelID,
String structureID)`
  
          Creates a Heterogen with the name and IDs given as arguments
(after adding the HETEROGEN\_PREFIX ("HET\_") to the beginning of
residueID).


| **Method Summary** | |
| --- | --- |
| `void` | `accept(Visitor visitor)`             Accepts a Visitor and does a callback. |
| `String` | `getName()`             Returns the Heterogen's name. |
| `String` | `processID(String id, String typeOfID)`             Overrides the processID() method of Residue in order to add guarantee that the residueID of a Heterogen always begins with the HETEROGEN\_PREFIX. |

**Methods inherited from class org.proteinshader.structure.Residue**

`addNewAtom, getAtom, getChainID, getModelID, getResidueID, getStructureID, iteratorAtoms, numberOfAtoms, toString`

**Methods inherited from class java.lang.Object**

`clone, equals, finalize, getClass, hashCode, notify, notifyAll, wait, wait, wait`

**Field Detail**

### HETEROGEN\_PREFIX

```
public static final String HETEROGEN_PREFIX
```

:   'HET\_' will be added to the beginning the residueID
    for a Heterogen (if it is not already present).

    **See Also:**: Constant Field Values


**Constructor Detail**

### Heterogen

```
public Heterogen(String name,
                 String residueID,
                 String chainID,
                 String modelID,
                 String structureID)
          throws MissingHetNameException,
                 InvalidIDException
```

:   Creates a Heterogen with the name and IDs given as arguments
    (after adding the HETEROGEN\_PREFIX ("HET\_") to the beginning of
    residueID). Any leading or trailing whitespace is trimmed from
    the residueID (before adding "HET\_") and from the name.

    **Parameters:**: `name` - Heterogen's name (preferably the full name).: `residueID` - ID of the Heterogen.: `chainID` - ID of the Chain.: `modelID` - ID of the Model.: `structureID` - ID of the Structure. **Throws:**: `MissingHetNameException` - if name is null or does not have at least one non-whitespace character before add the HETEROGEN\_PREFIX.: `InvalidIDException` - if residueID is null or does not have at least one non-whitespace character.


**Method Detail**

### accept

```
public void accept(Visitor visitor)
            throws VisitorException
```

:   Accepts a Visitor and does a callback.

    :   **Specified by:**: `accept` in interface `Visitable` **Specified by:**: `accept` in class `Residue`
    :   **Parameters:**: `visitor` - the Visitor to do a callback with. **Throws:**: `VisitorException` - if an error occurs while an object is being visited.

---


### getName

```
public String getName()
```

:   Returns the Heterogen's name.
    The String returned cannot be null or empty, because the
    constructor checks that this read-only attribute has at least
    one non-whitespace character.

    :   **Returns:**: The Heterogen name as a String.

---


### processID

```
public String processID(String id,
                        String typeOfID)
                 throws InvalidIDException
```

:   Overrides the processID() method of Residue in order to add
    guarantee that the residueID of a Heterogen always begins with
    the HETEROGEN\_PREFIX. If the HETEROGEN\_PREFIX is not already
    present, it will be added. Also, any leading or trailing
    whitespace will be trimmed off of the residueID.

    :   **Specified by:**: `processID` in interface `IDTest` **Overrides:**: `processID` in class `Residue`
    :   **Parameters:**: `id` - Residue ID to process.: `typeOfID` - type of ID (for possible use in error message). **Returns:**: The trimmed Residue ID beginning with the HETEROGEN\_PREFIX. **Throws:**: `InvalidIDException` - if the trimmed Residue ID does not have at least one character before adding the prefix.


---


|  |  |  |
| --- | --- | --- |
| **Overview** \| **Package** \| **Class** \| **Use** \| **Tree** \| **Deprecated** \| **Index** \| **Help** | |  |
| **PREV CLASS**   **NEXT CLASS** | **FRAMES**    **NO FRAMES**     **All Classes** |  |
| SUMMARY: NESTED \| FIELD \| CONSTR \| METHOD | DETAIL: FIELD \| CONSTR \| METHOD |  |


---

# *Copyright © 2007-2008*
